# Influence of High-Z Filler Chemistry on the Energy-Dependent Gamma-Ray Shielding of Cement-Board Composites

**DOI:** 10.3390/ma19194131

**Published:** 2026-09-27

**Authors:** Ahmed Alharbi

**Affiliations:** Department of Physics, College of Science, Qassim University, Buraydah 51452, Saudi Arabia; ahma.alharbi@qu.edu.sa

**Keywords:** cement-board composite, gamma-ray shielding, bismuth oxide, barium sulfate, hafnium oxide, tungsten trioxide, attenuation coefficient, half-value layer, X-ray diffraction

## Abstract

Cement-board composites were prepared at a fixed nominal filler loading to compare the attenuation response of different high-*Z* additives under a common mass-fraction condition. A commercial cement board was used as the host matrix, and five formulations containing 30 wt% Bi_2_O_3_, BaSO_4_, HfO_2_, WO_3_, or Fe were fabricated and compared with a pure pressed control. X-ray diffraction (XRD) was used to examine phase-related structural features, while gamma-ray transmission was measured with ^241^Am (59.5 keV) and ^137^Cs (661.7 keV) using a collimated NaI(Tl) spectrometric arrangement. At 59.5 keV, the linear attenuation coefficient increased from 0.3909 cm^−1^ for the control to 0.6423, 1.8127, 2.5464, 2.6205, and 2.7828 cm^−1^ for Fe-, HfO_2_-, WO_3_-, BaSO_4_-, and Bi_2_O_3_-containing specimens, respectively. The corresponding half-value layer decreased from 1.773 cm to 1.079, 0.382, 0.272, 0.265, and 0.249 cm. At 661.7 keV, the modified specimens remained more attenuating than the control but clustered within 0.1731–0.1842 cm^−1^. Within the tested conditions, the fabricated formulations showed much stronger discrimination at 59.5 keV than at 661.7 keV, with Bi_2_O_3_-, BaSO_4_-, and WO_3_-containing specimens forming the strongest low-energy group. Because the pressed-disc densities differed between the formulations, these linear-attenuation differences are interpreted as the combined response of additive identity and the resulting bulk density/compaction rather than as a chemistry-only effect. This study is a fixed-loading, two-energy screening comparison rather than a concentration or structural optimization study.

## 1. Introduction

The development of lead-free radiation-shielding materials increasingly focuses on composites that combine a processable host phase with dense or high-atomic-number constituents. Such systems can provide a useful compromise between photon attenuation, mass, manufacturability, cost, and environmental considerations. Cementitious matrices are particularly attractive because they are inexpensive, mechanically robust in their conventional form, and already used in building applications, while polymeric and hybrid systems offer complementary routes when flexibility or lower weight is required [1,2]. Conventional lead remains an efficient photon shield, but concerns related to toxicity and handling continue to motivate the investigation of alternative fillers and matrices [3].

The shielding response of a composite depends not only on density but also on photon energy and elemental composition. At tens of kiloelectronvolts, photoelectric absorption is strongly affected by atomic number and by the proximity of elemental absorption edges. Consequently, Bi-, Ba-, W-, and Hf-containing fillers can produce markedly different attenuation even when they are incorporated at the same mass fraction. At several hundred kiloelectronvolts, Compton scattering contributes more strongly and the dependence on elemental identity is reduced, so the performance of different heavy-element formulations often converges [4,5,6]. Evaluating the same set of materials at widely separated photon energies therefore provides a direct way to distinguish a composition-sensitive low-energy response from a more weakly composition-dependent intermediate-energy response.

A number of candidate fillers have been investigated in previous radiation-shielding studies. Bi_2_O_3_ has been used in Portland cement pastes, mortars, concrete, and polymer systems because it combines a high atomic number with the possibility of lead-free formulation [7,8,9]. BaSO_4_ is the principal dense component of barite-based shielding concrete and is widely used to increase photon attenuation [10]. HfO_2_ and WO_3_ have also shown favorable attenuation when dispersed in polymeric or composite matrices [11,12,13]. Recent work further shows that increasing density or incorporating ultrafine high-density constituents can improve cementitious shielding, although the accompanying changes in microstructure and mechanical behavior remain important [7,14]. Fe has a lower atomic number than Bi, W, Hf, and Ba, but its availability and comparatively low cost make it a useful lower-*Z* reference filler in a controlled comparison. The difficulty in comparing literature data directly is that published studies often differ simultaneously in host matrix, filler concentration, sample density, fabrication route, particle scale, photon energy, and measurement geometry. Consequently, literature values do not by themselves isolate the contribution of filler chemistry under otherwise common conditions.

The present work builds on, but is experimentally distinct from, an earlier study in which commercially available cement boards were characterized and their attenuation was measured before high-*Z* enhancement was explored using the NIST XCOM database and Geant4 calculations [15]. That previous study did not experimentally fabricate and measure the five 30 wt% filler-modified discs reported here. In the present study, the cement board is instead used as a common host material, and the comparison is performed under one preparation route, one nominal additive fraction, and one measurement geometry. This design reduces the confounding effects that arise when filler performance is inferred by comparing unrelated studies.

Accordingly, the objectives of this study were to: (i) fabricate a pure pressed cement-board control and five composites containing 30 wt% Bi_2_O_3_, BaSO_4_, HfO_2_, WO_3_, or Fe; (ii) compare their XRD profiles; (iii) measure the photopeak attenuation at 59.5 and 661.7 keV; and (iv) determine the linear and effective mass attenuation coefficients, half-value layer (HVL), tenth-value layer (TVL), and mean free path (MFP). The central contribution is a head-to-head experimental comparison of five filler-containing formulations at the same nominal mass fraction in the same cement-board matrix. Because the fillers differ in intrinsic density and compaction behavior, the comparison represents the experimentally realized formulation’s response rather than a chemistry-only effect independent of bulk density.

## 2. Materials and Methods

### 2.1. Materials and Specimen Preparation

A commercial cement board was mechanically reduced to a particulate form suitable for blending and pressing. Commercial Bi_2_O_3_, BaSO_4_, HfO_2_, WO_3_, and Fe powders were used as additives. Each modified formulation contained 70 wt% cement-board material and 30 wt% additive. For a nominal 4.0 g composite disc, 2.8 g of cement-board material and 1.2 g of additive were combined, homogenized, and compacted using a hydraulic press . Circular specimens with a diameter of approximately 2.5 cm were obtained. A pure cement-board disc was prepared using the same general pressing approach and served as the control.

The study was intentionally designed as a fixed-loading screening comparison rather than a concentration-optimization study. A nominal loading of 30 wt% was selected so that all five additives could be compared at the same relatively high filler fraction while retaining a common preparation route and self-supporting pressed-disc geometry. The purpose was therefore to hold the filler mass fraction constant so that the additive concentration was not a second independent variable. A fixed wt% does not, however, fix the filler volume fraction, specimen thickness, geometric density, or packing state. Because the additives differ in intrinsic density and compaction behavior, the measured linear attenuation represents the combined effect of additive identity and the bulk structure produced during pressing. Different loadings could change density, packing, particle contacts, porosity, and attenuation, so the present results should not be interpreted as identifying an optimum concentration.

For each modified specimen, the target mass fraction was defined gravimetrically as $w_{f}=m_{f}/\left(m_{f}+m_{c}\right)$, where $m_{f}$ and $m_{c}$ are the filler and cement-board masses, respectively. The nominal 1.2 g/2.8 g mixture gives $w_{f}=0.300$. The original fabrication record did not retain a calibrated balance uncertainty that would permit a defensible retrospective numerical uncertainty to be assigned to this nominal 30 wt% value. Accordingly, the revised manuscript reports the formulation as nominal and does not infer an unsupported precision from the displayed mass digits. If independent weighing uncertainties $\sigma_{m_{f}}$ and $\sigma_{m_{c}}$ are available, the corresponding loading uncertainty is obtained from standard propagation as

(1) $$\sigma_{w_{f}}=\frac{1}{{\left(m_{f}+m_{c}\right)}^{2}}\sqrt{m_{c}^{2}\sigma_{m_{f}}^{2}+m_{f}^{2}\sigma_{m_{c}}^{2}}.$$

The few-millimeter specimen thicknesses were not independently optimized for either photon energy. They resulted primarily from compacting a nominal 4.0 g charge in the same approximately 2.5 cm-diameter mold; the different final thicknesses therefore reflect differences in packing and geometric bulk density between the formulations. This approach preserved a measurable transmitted photopeak while producing mechanically handleable discs, but it also means that density and compaction are part of the experimentally realized material response. Particle-size distributions and particle morphologies of the commercial additives were not independently characterized, and quantitative filler dispersion was not verified during the original campaign; these factors are therefore treated as limitations rather than as controlled variables.

The measured specimen masses, diameters, and thicknesses are summarized in Table 1. The geometric bulk density of each pressed disc was calculated as

(2) $$\rho =\frac{m}{\pi {\left(d/2\right)}^{2}x},$$

where *m* is the specimen mass, *d* is the disc diameter, and *x* is the specimen thickness. The prepared specimens are shown in Figure 1.

### 2.2. Host-Matrix Baseline Composition and Nominal Formulation Chemistry

The elemental composition used as the baseline for the cement-board host was not treated as a new energy-dispersive X-ray spectroscopy (EDX) characterization in the present manuscript. The baseline corresponds to the previously published EDX composition of the CB4 commercial board reported by Alharbi [15]: C 10.99 wt%, O 51.44 wt%, Al 2.96 wt%, Si 11.51 wt%, S 0.45 wt%, Ca 19.98 wt%, Fe 1.20 wt%, and Cu 1.47 wt%; Mg was not detected. Those previously reported data are used here only to estimate nominal elemental fractions after addition of 30 wt% of each filler. The modified-specimen values in Table 2 are therefore formulation-based estimates and are not presented as new EDX measurements of the composite discs.

For additional compositional context, a simple atom-fraction-weighted mean atomic number was calculated as $\bar{Z}=\sum_{i}x_{i}Z_{i}$, where $x_{i}$ is the atomic fraction derived from the nominal elemental composition and $Z_{i}$ is the atomic number. The resulting descriptive values are 9.56 for the host baseline, 11.87 for Bi_2_O_3_, 10.92 for BaSO_4_, 11.64 for HfO_2_, 11.42 for WO_3_, and 11.68 for Fe formulations. These values are included only as a composition descriptor; they are not an effective atomic number, $Z_{\mathrm{eff}}$, because photon attenuation is energy dependent and cannot be represented rigorously by a single arithmetic average of atomic number. The strong 59.5 keV differences observed between samples with similar $\bar{Z}$ also illustrate why absorption-edge position, compound stoichiometry, and density must be considered in addition to a simple average-*Z* metric.

### 2.3. X-Ray Diffraction

X-ray diffraction was performed on the pure pressed cement-board specimen and on all five filler-containing composites using a Cu-target diffractometer with Cu Kα radiation. The tube was operated at 40 kV and 30 mA. Data were collected in conventional coupled θ–2θ geometry, in which the incident and diffracted angles are varied symmetrically with respect to the specimen surface while the detector records intensity as a function of 2θ. The scan covered 2θ = 10–80° using continuous scanning at 2.0° min^−1^, a sampling interval of 0.020°, and a preset time of 0.60 s per point. The patterns were normalized individually to their maximum intensity and vertically offset to facilitate comparison. Phase-related peak assignments were restricted to reflections that could be associated with the filler-containing patterns with reasonable confidence. The Bi_2_O_3_, BaSO_4_, and HfO_2_ reflections were treated as direct phase-related assignments, whereas the WO_3_ and Fe annotations were used more cautiously as reference-supported tentative assignments because of matrix overlap and the comparatively high background of those scans.

### 2.4. Gamma-Ray Transmission Measurements

Transmission measurements were carried out using sealed ^241^Am (59.5 keV, 370 kBq) and ^137^Cs (661.7 keV, 3.7 MBq) point sources and a BICRON 2M2/2 NaI(Tl) scintillation detector with a 2-inch × 2-inch crystal. A lead collimator approximately 10 cm × 10 cm in face dimensions and 5 cm thick was used to restrict the beam. The aperture was tapered from 1.0 cm at the entrance to 0.5 cm at the exit. The specimen was placed approximately 5 cm from the source and 15 cm from the detector, and the geometry was kept fixed for the unshielded and shielded measurements. Each spectrum used in the attenuation analysis was collected for 600 s. Figure 2 shows the measurement arrangement.

For each spectrum, a region of interest was selected around the full-energy photopeak at 59.5 or 661.7 keV. The photopeak was fitted with a Gaussian function and the integrated fitted counts were used as $I_{0}$ for the unshielded acquisition and *I* for the corresponding shielded acquisition. Consequently, the reported attenuation parameters are based on photopeak transmission rather than total-spectrum counts. The same ROI and fitting procedure was applied consistently within each source dataset. At 661.7 keV, one unshielded reference spectrum was used for the set of measurements acquired in the unchanged geometry; hence, the same $I_{0}$ is used for the corresponding specimen calculations reported below. This common reference does not constitute repeated independent measurements of $I_{0}$: its counting uncertainty is shared, and therefore correlated, among the attenuation coefficients derived from that reference. At 59.5 keV, the WO_3_ run used a separate matched unshielded reference, as stated with the corresponding results.

The linear attenuation coefficient was calculated from the Beer–Lambert relation:

(3) $$\mu =\frac{\ln(I_{0}/I)}{x},$$

where *x* is the specimen thickness in centimeters. An effective mass attenuation coefficient was then calculated from the geometric bulk density:

(4) $$\mu_{m}=\frac{\mu }{\rho }.$$

Because ρ is the geometric density of the pressed disc, the resulting $\mu_{m}$ values are described here as effective mass-normalized coefficients for the tested specimens rather than as independently measured elemental cross-sections.

The half-value layer, tenth-value layer, and mean free path were obtained from

(5) $$\mathrm{HVL}=\frac{\ln2}{\mu },\;\mathrm{TVL}=\frac{\ln10}{\mu },\;\mathrm{MFP}=\frac{1}{\mu }.$$

### 2.5. Uncertainty and Reproducibility

For the photopeak counts, Poisson statistics were assumed, $\sigma_{N}=\sqrt{N}$. Propagation through Equation (3) gives the counting-statistical component of the uncertainty in μ as

(6) $$\sigma_{\mu ,\mathrm{count}}=\frac{1}{x}\sqrt{{\left(\frac{\sigma_{I_{0}}}{I_{0}}\right)}^{2}+{\left(\frac{\sigma_{I}}{I}\right)}^{2}}.$$

If an independently established thickness uncertainty $\sigma_{x}$ is available, the corresponding first-order combined uncertainty in μ is

(7) $$\sigma_{\mu }=\sqrt{\frac{1}{x^{2}}\left[{\left(\frac{\sigma_{I_{0}}}{I_{0}}\right)}^{2}+{\left(\frac{\sigma_{I}}{I}\right)}^{2}\right]+{\left(\frac{\mu \sigma_{x}}{x}\right)}^{2}}.$$

Likewise, for the geometric density in Equation (2), first-order propagation gives

(8) $${\left(\frac{\sigma_{\rho }}{\rho }\right)}^{2}={\left(\frac{\sigma_{m}}{m}\right)}^{2}+{\left(2\frac{\sigma_{d}}{d}\right)}^{2}+{\left(\frac{\sigma_{x}}{x}\right)}^{2}.$$

The numerical error bars shown in this study continue to represent counting statistics only. The original experimental record does not contain a complete set of calibrated instrument uncertainties for mass, diameter, and thickness that would justify reconstructing numerical systematic error bars after the fact. Rather than assigning unsupported values, possible systematic contributions are explicitly identified as thickness and diameter measurement, sample repositioning relative to the collimated beam, compaction and density estimation, Gaussian ROI fitting, detector/source stability, and material heterogeneity. The use of a common unshielded reference also introduces covariance among attenuation coefficients calculated from that reference. Repeat acquisitions existed for selected configurations but not for the full specimen-by-energy matrix; consequently, a replicate-based standard deviation is not reported for the complete dataset. This limitation is especially relevant at 661.7 keV, where the modified specimens differ from one another by only about 0.011 cm^−1^, and therefore the small ordering among those five formulations should not be over-interpreted. At 59.5 keV, the separation between the high-attenuation group (Bi_2_O_3_, BaSO_4_, and WO_3_) and HfO_2_/Fe/control is much larger than the quoted counting-statistical errors, but the absence of a complete numerical systematic budget means that close pairwise differences within the high-attenuation group, particularly BaSO_4_ versus WO_3_, are interpreted cautiously rather than as a definitive rank.

## 3. Results

### 3.1. XRD Characteristics of the Pressed Composites

The normalized XRD patterns are presented in Figure 3. The pure pressed cement board shows the diffraction features of the host matrix, whereas several filler-containing specimens develop additional reflections or marked changes in relative peak intensity. The most readily resolved filler-related features are observed for the Bi_2_O_3_-, BaSO_4_-, and HfO_2_-containing specimens.

For the Bi_2_O_3_ composite, reflections near 27.58°, 33.64°, 47.16°, and 58.58° are consistent with monoclinic α-Bi_2_O_3_ and are assigned to the (120), (122), (041), and (024) planes, respectively, in agreement with commonly reported Bi_2_O_3_ diffraction features in shielding composites [7,8]. The BaSO_4_ specimen displays the characteristic multi-peak barite pattern; selected reflections near 23.56°, 29.48°, 32.24°, and 43.24° are shown in Figure 3, with additional observed reflections listed in Table 3. For HfO_2_, the observed pattern contains reflections consistent with monoclinic HfO_2_, including features near 28.94°, 32.18°, 35.06°, 50.78°, and 60.36°.

The WO_3_-containing pattern differs clearly from the pure-board pattern and contains several visible maxima that coincide with expected WO_3_ reference positions. In Figure 3, selected WO_3_ features are therefore annotated at approximately 28.94°, 41.52°, and 72.44° as tentative/reference-supported assignments rather than as definitive phase confirmation. The Fe-containing pattern exhibits a comparatively high background, but features near 44.67° and 65.02° coincide with the expected bcc α-Fe (110) and (200) positions. These Fe annotations are likewise treated as tentative because the corresponding peaks are not as clearly resolved as those observed for Bi_2_O_3_, BaSO_4_, and HfO_2_. Throughout the manuscript, the labels “WO_3_-containing” and “Fe-containing” identify the nominal commercial additives introduced during specimen preparation; they do not imply that XRD independently established phase purity. The attenuation comparisons therefore refer to the known formulation labels, while the stated XRD confidence applies only to the diffraction-based phase assignments.

### 3.2. Attenuation at 661.7 keV

The measured ^137^Cs transmission data are summarized in Table 4. The pure pressed cement board yielded μ=0.1248±0.0044 cm^−1^. Every filler-containing specimen produced a larger coefficient, ranging from 0.1731 cm^−1^ for Fe to 0.1842 cm^−1^ for BaSO_4_. Thus, the filler addition increased attenuation relative to the control, but the five modified specimens occupied a narrow interval of only 0.0111 cm^−1^. Considering the quoted counting-statistical uncertainties and the unquantified systematic contributions discussed in Section 2.5, the differences among the modified formulations at 661.7 keV should be regarded as small; the robust observation is their common improvement relative to the pure control rather than a precise rank ordering of the five fillers. The corresponding linear attenuation coefficients are shown in Figure 4.

### 3.3. Attenuation at 59.5 keV

A much larger separation among the fabricated formulations is observed for ^241^Am (Table 5). The control value was 0.3909 cm^−1^, increasing to 0.6423 cm^−1^ for Fe, 1.8127 cm^−1^ for HfO_2_, 2.5464 cm^−1^ for WO_3_, 2.6205 cm^−1^ for BaSO_4_, and 2.7828 cm^−1^ for Bi_2_O_3_. Relative to the pure pressed board, these values correspond to increases of approximately 64%, 364%, 551%, 570%, and 612%, respectively. Based on the measured central values, Bi_2_O_3_, BaSO_4_, and WO_3_ form the strongest low-energy group, followed by HfO_2_, Fe, and the pure cement board. Because the error bars represent counting statistics only, the close pairwise ordering within the top group is not treated as definitive. The corresponding linear attenuation coefficients are shown in Figure 5.

For the WO_3_ specimen, a separate matched unshielded reference acquisition was used ($I_{0}$ = 390,605 counts), whereas the remaining ^241^Am measurements used $I_{0}$ = 376,739 counts. The use of the matched reference is retained explicitly in Table 5 so that the attenuation calculation can be reproduced directly.

### 3.4. Effective Mass Attenuation Coefficient and Penetration Parameters

The effective mass attenuation coefficients, HVLs, TVLs, and MFPs derived from the measured μ values and geometric specimen densities are listed in Table 6. At 59.5 keV, the HVL decreases from 1.773 cm for the control to 1.079 cm for Fe and 0.382 cm for HfO_2_, and reaches 0.272, 0.265, and 0.249 cm for WO_3_, BaSO_4_, and Bi_2_O_3_, respectively. This corresponds to an HVL reduction of approximately 85–86% for the three strongest low-energy formulations relative to the pure pressed board. The MFP values follow the same inverse dependence on μ.

At 661.7 keV, the modified specimens have HVLs of 3.763–4.004 cm compared with 5.554 cm for the control and MFPs of 5.429–5.777 cm compared with 8.013 cm. The effective mass-normalized coefficients are much more tightly grouped at this energy. Because $\mu_{m}$ is normalized by the geometric density of each pressed specimen, its ranking is not expected to reproduce exactly the ranking of μ. The corresponding HVL and MFP trends are illustrated in Figure 6 and Figure 7, respectively.

## 4. Discussion

### 4.1. Energy Dependence of Filler Performance

The most important result is the change in material discrimination between 59.5 and 661.7 keV. At 59.5 keV, the response separates the Bi_2_O_3_/BaSO_4_/WO_3_ high-attenuation group from HfO_2_, Fe, and the control, while the central values within the top group are closer to one another. At 661.7 keV, all five filler-containing formulations still improve μ relative to the pure board, but their coefficients differ only slightly. This contrast is consistent with the transition from a strongly composition-sensitive low-energy regime, in which photoelectric absorption is important, to an intermediate-energy regime in which Compton scattering contributes much more strongly [4,5,6]. Because only two photon energies were measured, the present data do not establish a continuous ranking between them. In the 100–500 keV interval, the K edges of Ba, Hf, W, and Bi all lie below or near the lower boundary of this range (approximately 37.45, 65.35, 69.53, and 90.54 keV, respectively), while the photoelectric contribution decreases rapidly with increasing energy and Compton scattering becomes progressively more important [16]. A general convergence of formulation-specific differences is therefore physically plausible, but the exact ordering at intermediate energies is not inferred from the two measured points.

The low-energy ranking is not determined by atomic number alone. The attenuation of a compound depends on its full elemental composition, density, and photon energy, including the position of relevant absorption edges. At 59.5 keV, the photon energy lies above the Ba K-edge (approximately 37.45 keV) but below the Hf, W, and Bi K edges (approximately 65.35, 69.53, and 90.54 keV, respectively) [16]. This edge placement, together with compound stoichiometry and specimen density, helps explain why BaSO_4_ can slightly exceed WO_3_ in the measured linear coefficient despite tungsten having the larger atomic number. The Bi_2_O_3_ formulation gives the largest μ per unit thickness, whereas BaSO_4_ gives the largest effective $\mu_{m}$ among the modified specimens at 59.5 keV. These two rankings are not contradictory because μ contains the experimentally realized bulk-density contribution whereas $\mu_{m}$ additionally normalizes by geometric density. The comparison also demonstrates why neither elemental *Z* nor the simple $\bar{Z}$ descriptor introduced in Section 2.2 can predict the low-energy response by itself.

Fe provides a useful lower-*Z* comparison. Its 30 wt% addition increases μ by about 64% at 59.5 keV and 39% at 661.7 keV relative to the pure pressed control, but its low-energy performance remains well below that of the Bi-, Ba-, W-, and Hf-containing formulations. This result illustrates the value and the limit of a fixed-loading design: it removes additive mass fraction as a varying parameter, while the measured response still contains the effects of additive identity, density, packing, and compaction in the pressed disc.

Density and compaction contribute materially to the measured linear attenuation. A common 30 wt% fixes the additive mass fraction but does not fix filler volume fraction or the bulk structure of the pressed disc. Relative to the 1.494 g cm^−3^ control, the modified-disc densities span 1.680–2.063 g cm^−3^ and their thicknesses span 3.95–4.85 mm. Accordingly, μ is interpreted as a formulation-level quantity that includes both composition and the experimentally realized density/packing state. Comparing μ with $\mu_{m}$ helps separate these effects qualitatively. At 59.5 keV, for example, Bi_2_O_3_ has the largest linear coefficient (2.7828 cm^−1^), whereas BaSO_4_ has the largest effective mass-normalized coefficient (1.4470 cm^2^ g^−1^). At 661.7 keV, the linear coefficients are tightly clustered, although the density-normalized values span 0.0884–0.1030 cm^2^ g^−1^ among the modified specimens. Thus, density and compaction affect attenuation per unit thickness, while the change in ranking after density normalization also shows that density alone does not account for the low-energy behavior.

### 4.2. Comparison with Published Cementitious Shielding Materials

Table 7 provides context for the 661.7 keV data. The modified cement-board specimens, with μ = 0.1731–0.1842 cm^−1^, overlap the 0.172–0.195 cm^−1^ range reported for ordinary concrete of different grades [10]. They exceed the values of 0.146–0.160 cm^−1^ reported for ordinary concrete with siltstone replacement [17], while remaining below the 0.209–0.230 cm^−1^ values reported for conventional and Bi_2_O_3_-modified concrete in another study [9] and below the 0.2266 cm^−1^ reported for a high-density barite/steel-slag concrete [14]. These literature values should not be interpreted as a direct ranking because the compared materials differ in density, geometry, composition, fabrication, and experimental method.

Conventional lead remains substantially more efficient per unit thickness and is included here as a benchmark rather than as a directly competing cementitious material. Interpolation of the NIST elemental-lead attenuation data gives approximate mass attenuation coefficients of 5.13 cm^2^ g^−1^ at 59.5 keV and 0.111 cm^2^ g^−1^ at 661.7 keV. Using the NIST lead density of approximately 11.35 g cm^−3^ gives corresponding linear coefficients of about 58.2 and 1.26 cm^−1^ and HVLs of approximately 0.012 and 0.55 cm, respectively [16]. These HVL values are much smaller than for the present composites. The motivation for the cement-board systems is therefore not to claim superior attenuation to lead at equal thickness, but to explore lead-free, lower-density, cement-compatible formulations that may be useful where toxicity, handling, integration into building products, or distributed shielding thickness are important.

The present study should also be distinguished from the author’s earlier cement-board publication [15]. The earlier work compared commercial boards in their supplied form and used simulations to explore possible high-*Z* enhancement. The current attenuation values were obtained from newly prepared pressed discs containing one filler at a time at a fixed 30 wt% loading. The pure control was also reprocessed and pressed; therefore, its density and attenuation value should not be treated as a repeat measurement of the earlier commercial slab.

### 4.3. Scope, Limitations, and Material Implications

The results establish an attenuation comparison under one controlled formulation condition rather than an optimized construction material. Only one nominal filler loading and two photon energies are represented in the dataset, so the conclusions are restricted to those conditions and no continuous ranking is assigned across diagnostic, industrial, or nuclear photon-energy spectra. In the intermediate-energy region the photoelectric contribution decreases while Compton scattering becomes more important, and above 1.022 MeV pair production becomes energetically possible and grows in relative importance with photon energy and high-*Z* content. The 2.614 MeV ^208^Tl line is therefore outside the scope of the present dataset, and no high-energy ranking is inferred here.

Particle size, morphology, and filler dispersion can influence packing density, porosity, interfacial structure, and local path-length distribution. These variables were not independently quantified here. The XRD data show phase-related features most clearly for Bi_2_O_3_, BaSO_4_, and HfO_2_, whereas the WO_3_ and Fe scans provide only tentative/reference-supported assignments because of matrix overlap and, particularly for Fe, a comparatively high background. The specimen labels refer to the nominal commercial additives used in fabrication and are distinct from the confidence assigned to XRD phase identification. XRD alone does not establish spatially homogeneous dispersion. SEM imaging, EDS elemental mapping, and particle-size analysis are recognized as complementary microstructural characterization methods, but they are outside the present dataset. The conclusions therefore concern the measured bulk attenuation of the fabricated discs and do not assume perfectly homogeneous microscopic dispersion or XRD-confirmed phase purity for WO_3_ and Fe.

The incorporation of 30 wt% high-density powder can also alter material properties that were outside the scope of this attenuation-screening study. Changes in porosity, hydration, particle packing, interfacial bonding, and water demand may influence compressive or flexural strength, water uptake, durability, and dimensional stability; such effects have been reported as relevant considerations in high-*Z* cementitious systems [7]. These properties are not evaluated in the present dataset, and no claim is made regarding structural, durability, or building-code performance.

Monte Carlo or database calculations could provide complementary physical context. XCOM could generate composition-dependent mass attenuation coefficients across a continuous energy grid, while Geant4 or MCNP could represent the finite collimator, specimen, detector, and scattering geometry and separately score transmitted, scattered, and secondary-photon components. The present paper, however, is intentionally based on measured transmission at 59.5 and 661.7 keV, and its reported attenuation coefficients and conclusions do not depend on a simulation result.

The present work examined single-filler formulations so that each additive identity could be considered without an additional compositional variable. Hybrid systems such as Bi_2_O_3_/WO_3_ or Bi_2_O_3_/BaSO_4_ may be attractive because they combine constituents with different absorption-edge locations, densities, costs, and processing behavior. A broader energy response is physically plausible, but the present single-filler dataset does not support a claim of synergistic behavior and no such claim is made.

Within these limits, the controlled fixed-loading comparison demonstrates that additive selection can substantially reduce the thickness associated with low-energy photon attenuation. Bi_2_O_3_-, BaSO_4_-, and WO_3_-containing specimens reduce the 59.5 keV HVL from 1.773 cm to approximately 0.25–0.27 cm, while HfO_2_ provides an intermediate response and Fe a more modest enhancement. This is interpreted as an attenuation-screening result for the stated loading, specimen preparation, and photon energies.

## 5. Conclusions

A controlled experimental screening comparison was performed using a common cement-board host and a fixed nominal 30 wt% addition of Bi_2_O_3_, BaSO_4_, HfO_2_, WO_3_, or Fe. Under the tested conditions, additive identity together with the resulting pressed-disc density and compaction had a strong influence on the measured linear attenuation at 59.5 keV: μ increased from 0.3909 cm^−1^ for the pure pressed control to 2.7828, 2.6205, and 2.5464 cm^−1^ for Bi_2_O_3_, BaSO_4_, and WO_3_, respectively, with corresponding HVLs of 0.249, 0.265, and 0.272 cm. HfO_2_ also produced a substantial improvement, whereas Fe gave a smaller enhancement. At 661.7 keV, all five modified specimens remained more attenuating than the control, but their coefficients clustered between 0.1731 and 0.1842 cm^−1^. Given the narrow separation and the fact that the plotted uncertainties represent counting statistics rather than a complete systematic budget, a precise ordering of the five modified formulations at 661.7 keV is not claimed.

XRD provided clear phase-related features for the Bi_2_O_3_-, BaSO_4_-, and HfO_2_-containing composites. Selected WO_3_ and Fe features were annotated only as tentative/reference-supported assignments because of matrix overlap and the comparatively high background of the corresponding scans. Within the specific combination of one nominal loading and two measured photon energies, Bi_2_O_3_-, BaSO_4_-, and WO_3_-containing specimens formed the strongest attenuation group at 59.5 keV. Because a complete numerical systematic budget is unavailable, the close pairwise ordering within this top group is not treated as definitive. This result should not be interpreted as identifying a universally optimal shielding formulation. Concentration dependence, continuous energy response, microscopic dispersion, structural/durability performance, and hybrid-filler synergy are outside the scope of the present dataset, and no conclusions are drawn about those properties.

## Figures and Tables

**Figure 1:**
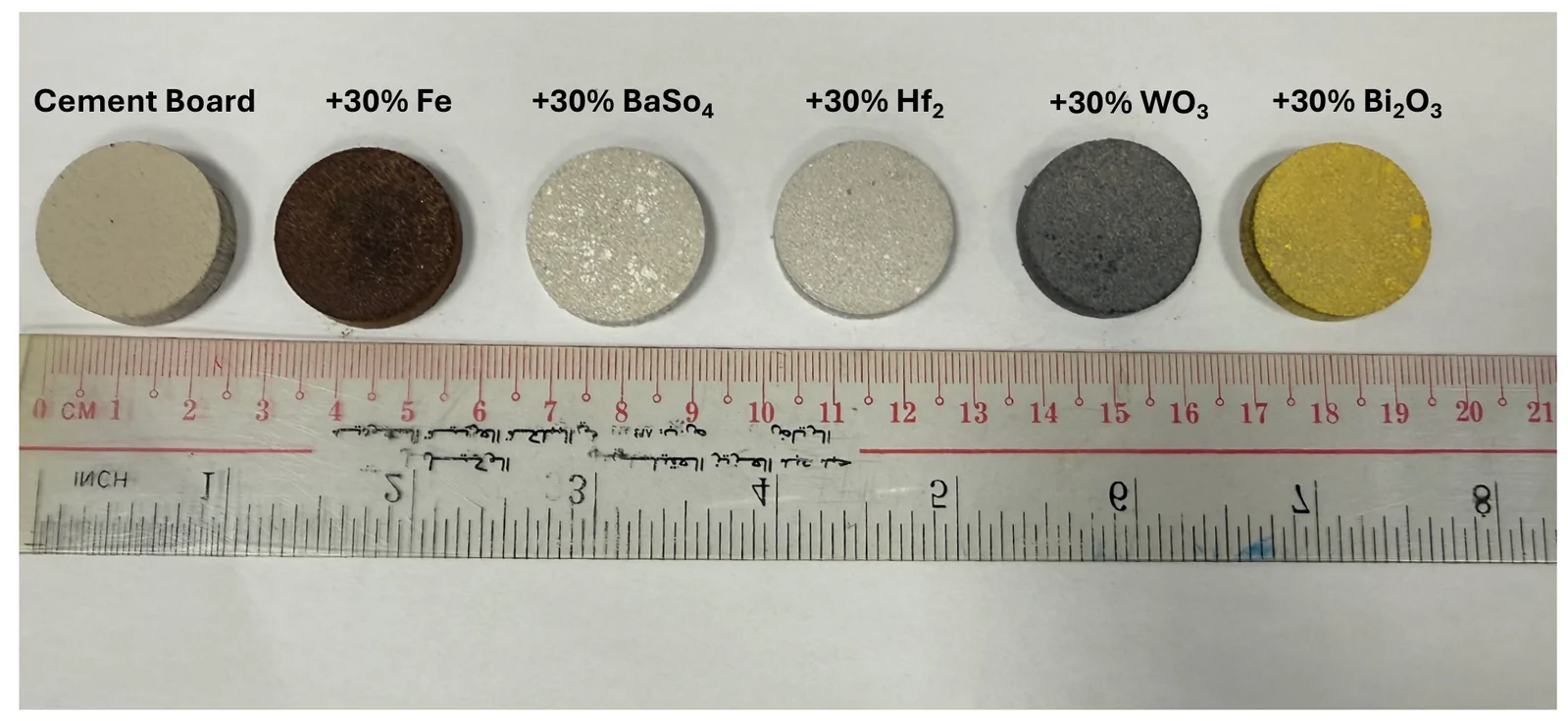
Pressed specimens used in the study. From left to right: pure cement board, cement board + 30 wt% Fe, +30 wt% BaSO_4_, +30 wt% HfO_2_, +30 wt% WO_3_, and +30 wt% Bi_2_O_3_. The Arabic markings visible on the ruler are scale annotations only and do not denote sample identities or experimental variables.

**Figure 2:**
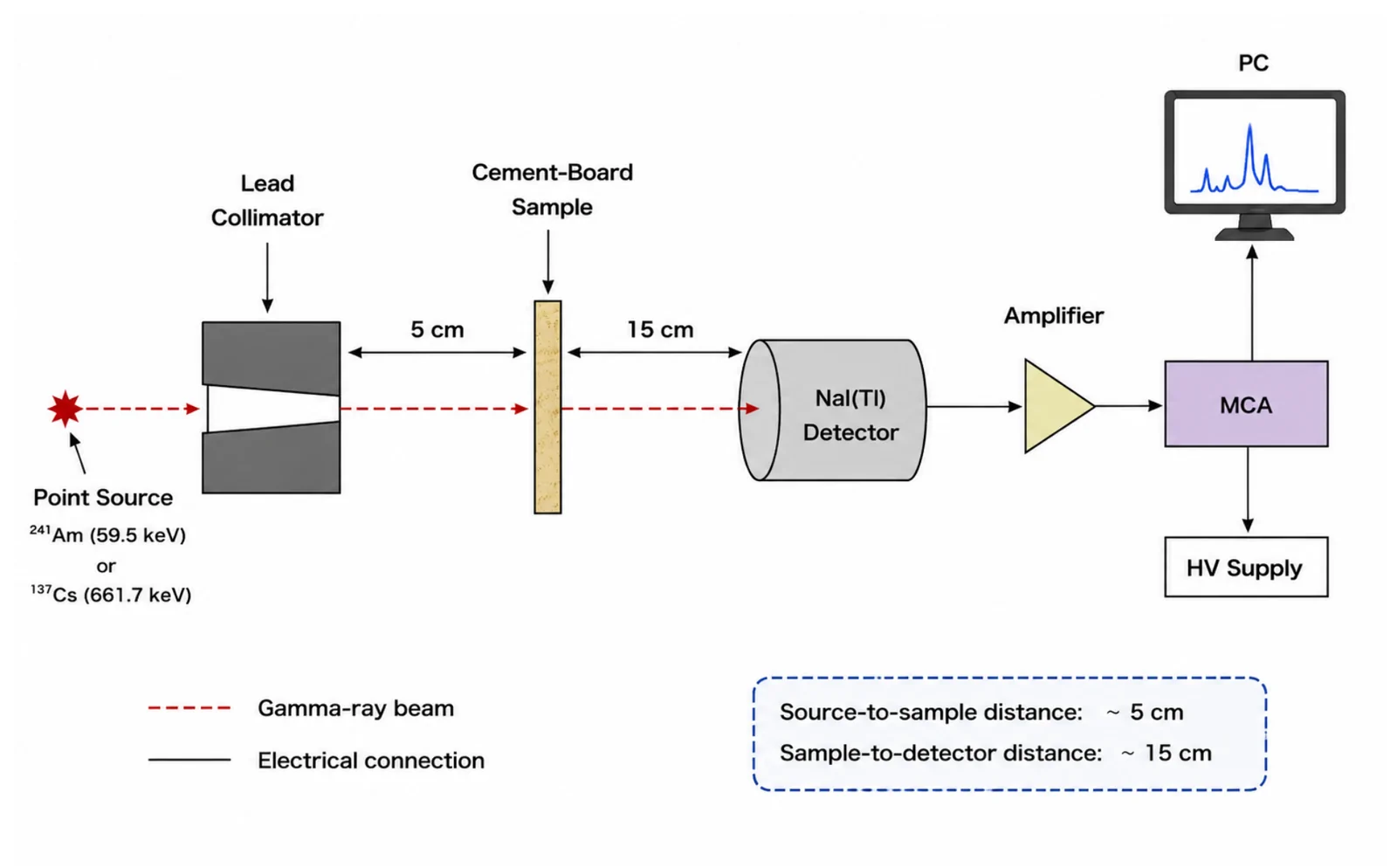
Schematic of the collimated transmission arrangement used with ^241^Am and ^137^Cs. The specimen was positioned approximately 5 cm from the source and 15 cm from the NaI(Tl) detector. Each attenuation spectrum was acquired for 600 s.

**Figure 3:**
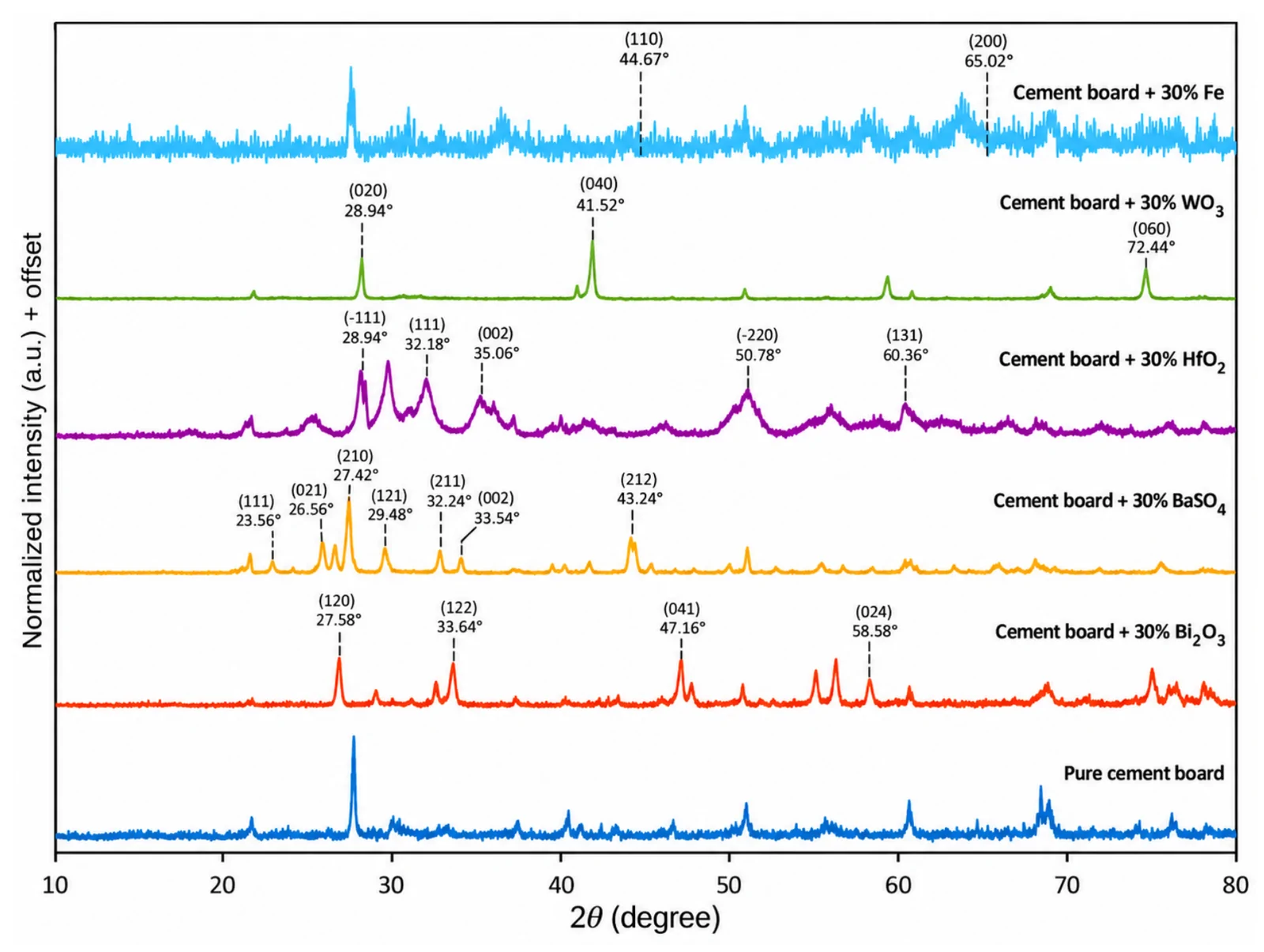
Normalized and vertically offset XRD patterns of the pure pressed cement board and composites containing 30 wt% Bi_2_O_3_, BaSO_4_, HfO_2_, WO_3_, or Fe. Selected reflections are labeled for comparison. The assignments for Bi_2_O_3_, BaSO_4_, and HfO_2_ correspond to clearly resolved phase-related reflections. The WO_3_ and Fe annotations are shown as tentative/reference-supported assignments because of overlap with the cement-board matrix and the comparatively high background of the corresponding scans.

**Figure 4:**
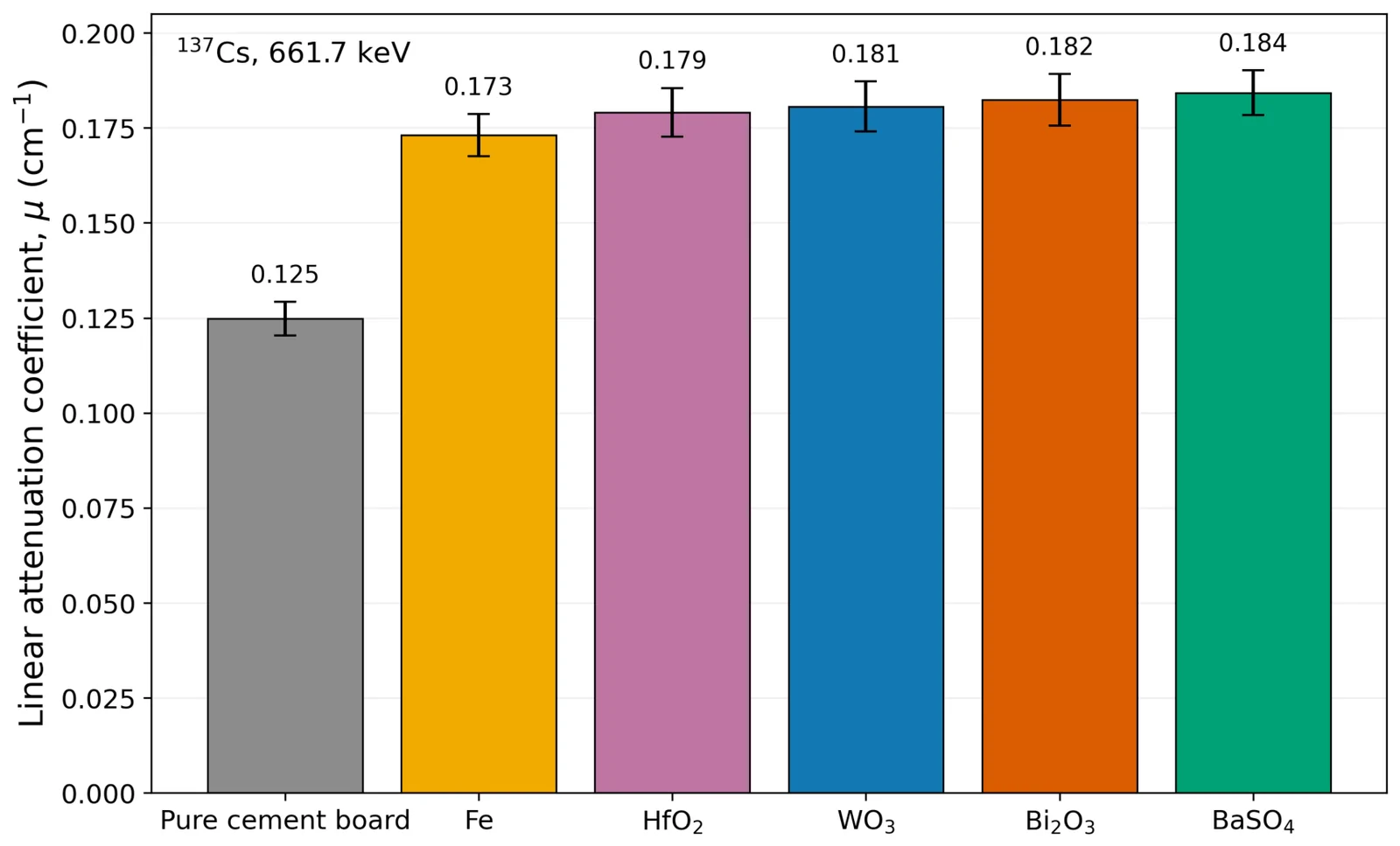
Linear attenuation coefficients measured at 661.7 keV. Error bars represent counting-statistical uncertainty only.

**Figure 5:**
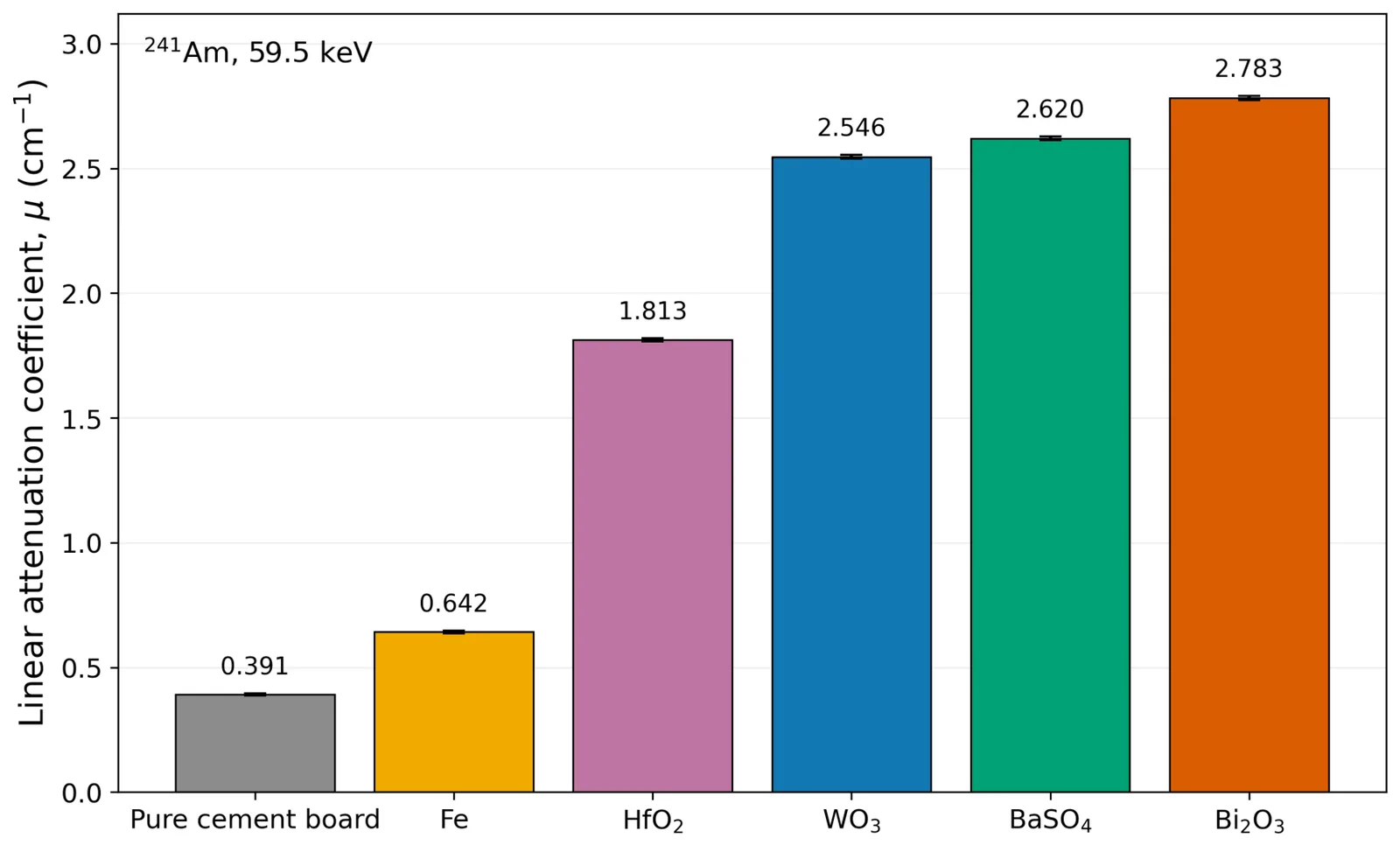
Linear attenuation coefficients measured at 59.5 keV. Error bars represent counting-statistical uncertainty only.

**Figure 6:**
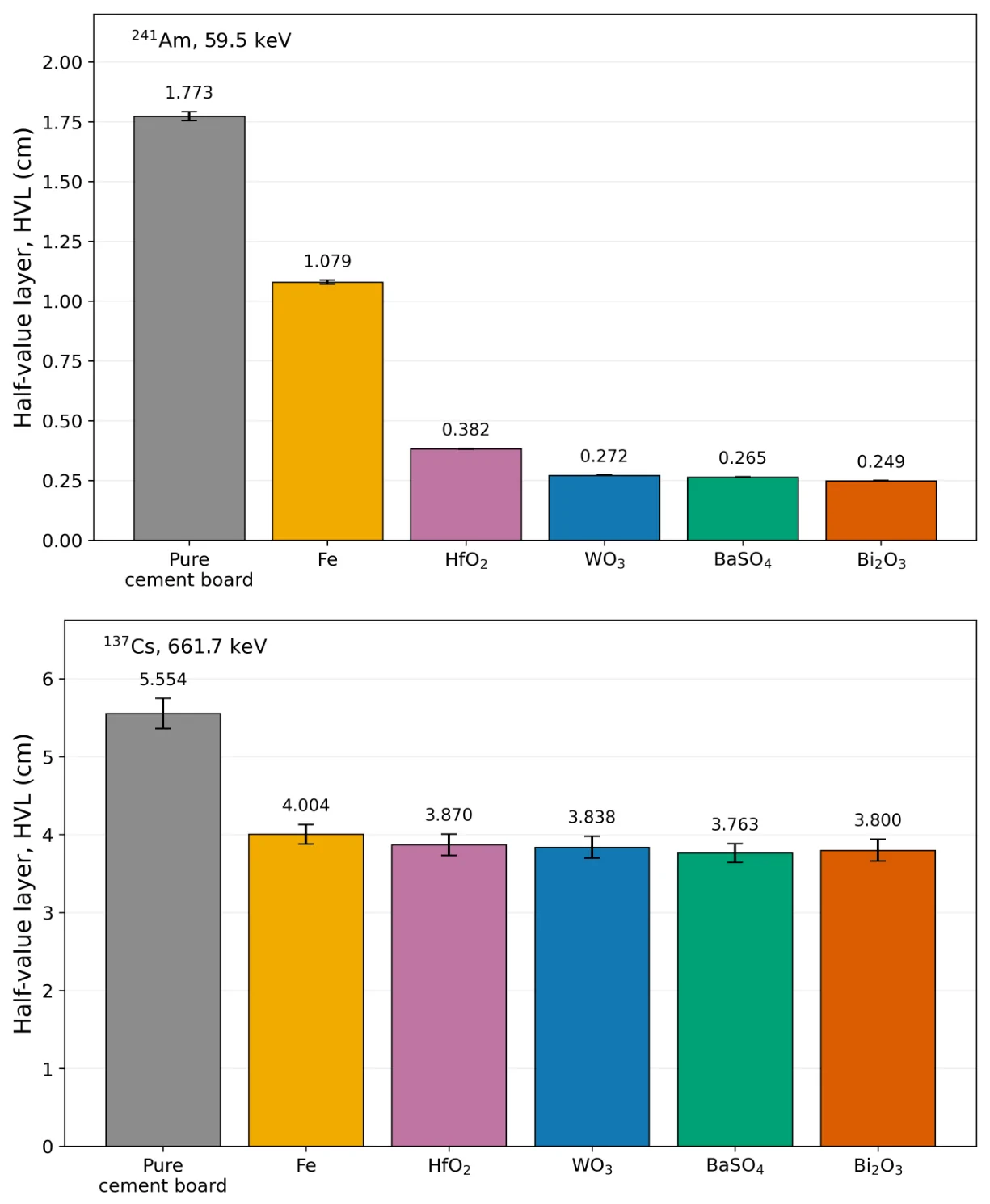
Half-value layer derived from the measured attenuation coefficients at 59.5 and 661.7 keV. A lower HVL indicates greater attenuation per unit thickness. Error bars reflect propagation of counting-statistical uncertainty in μ only.

**Figure 7:**
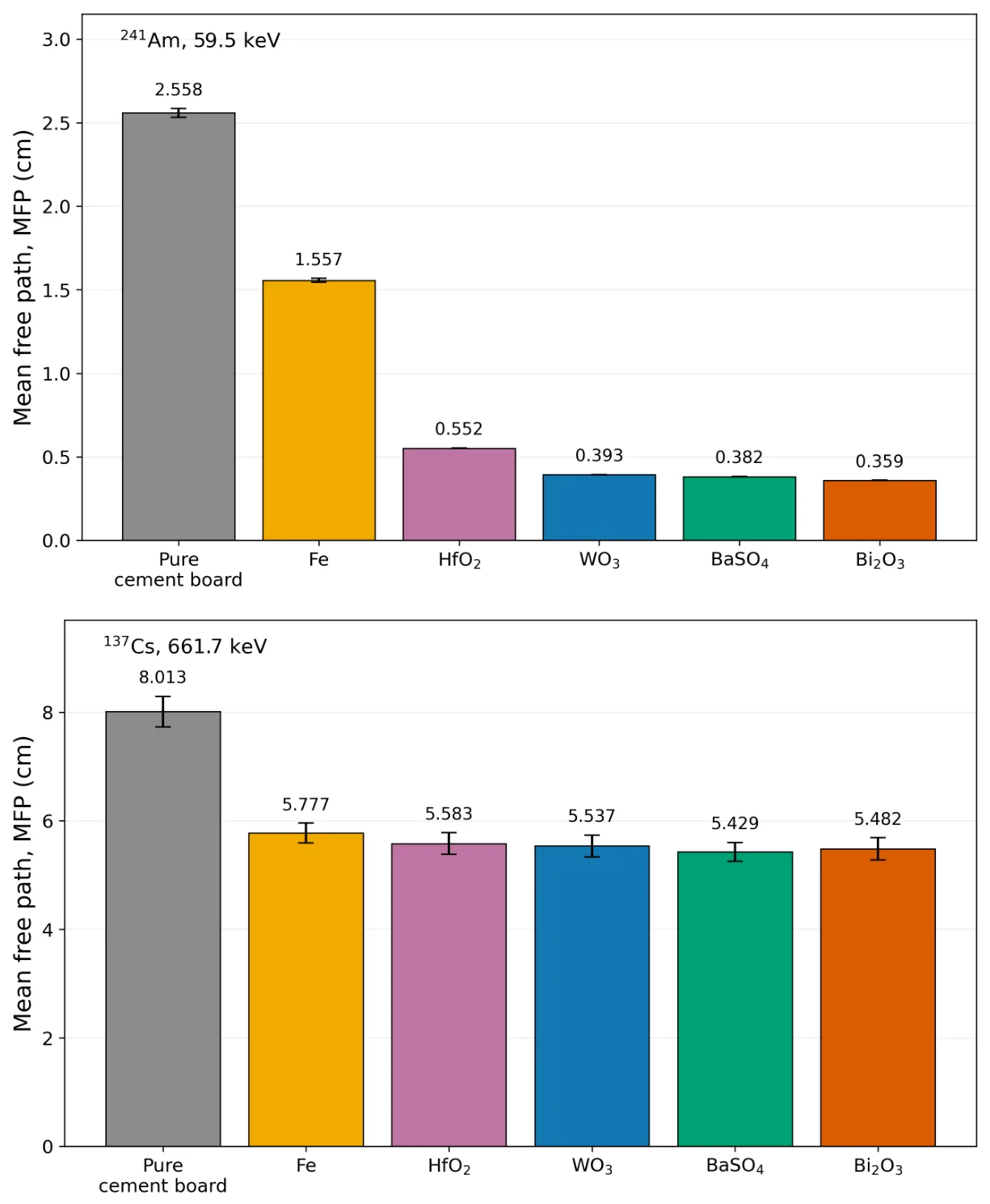
Mean free path derived from the measured attenuation coefficients at 59.5 and 661.7 keV. A lower MFP corresponds to a shorter average photon travel distance between interactions. Error bars reflect propagation of counting-statistical uncertainty in μ only.

**Table 1:** Formulation, geometry, and geometric bulk density of the pressed specimens.

Specimen	Mass (g)	Cement Board/Additive (wt%)	Diameter (cm)	Thickness (mm)	Density (g cm^−3^)
Pure cement board	4.40	100/0	2.50	6.00	1.494
Cement board + 30% Bi_2_O_3_	4.00	70/30	2.50	3.95	2.063
Cement board + 30% BaSO_4_	4.00	70/30	2.50	4.50	1.811
Cement board + 30% HfO_2_	4.00	70/30	2.50	4.15	1.964
Cement board + 30% WO_3_	4.00	70/30	2.50	4.04	2.017
Cement board + 30% Fe	4.00	70/30	2.50	4.85	1.680

**Table 2:** Previously reported host-board baseline composition [15] and nominal elemental composition of the 30 wt% filler formulations. Values are in wt%.

Sample	C	O	Mg	Al	Si	S	Ca	Fe	Cu	Bi	Ba	Hf	W
Host baseline (previously reported)	10.990	51.440	0.000	2.960	11.510	0.450	19.980	1.200	1.470	0	0	0	0
+30% Bi_2_O_3_	7.693	39.098	0.000	2.072	8.057	0.315	13.986	0.840	1.029	26.910	0	0	0
+30% BaSO_4_	7.693	44.234	0.000	2.072	8.057	4.437	13.986	0.840	1.029	0	17.652	0	0
+30% HfO_2_	7.693	40.569	0.000	2.072	8.057	0.315	13.986	0.840	1.029	0	0	25.439	0
+30% WO_3_	7.693	42.219	0.000	2.072	8.057	0.315	13.986	0.840	1.029	0	0	0	23.789
+30% Fe	7.693	36.008	0.000	2.072	8.057	0.315	13.986	30.840	1.029	0	0	0	0

**Table 3:** Representative XRD reflections used for the phase-related assignments in Figure 3.

Specimen	2θ (deg)	(hkl)	Assignment
Bi_2_O_3_	27.58	(120)	Monoclinic α-Bi_2_O_3_
	33.64	(122)	Monoclinic α-Bi_2_O_3_
	47.16	(041)	Monoclinic α-Bi_2_O_3_
	58.58	(024)	Monoclinic α-Bi_2_O_3_
BaSO_4_	23.56	(111)	Orthorhombic barite BaSO_4_
	26.56	(021)	Orthorhombic barite BaSO_4_
	27.42	(210)	Orthorhombic barite BaSO_4_
	29.48	(121)	Orthorhombic barite BaSO_4_
	32.24	(211)	Orthorhombic barite BaSO_4_
	33.54	(002)	Orthorhombic barite BaSO_4_
	43.24	(212)	Orthorhombic barite BaSO_4_
HfO_2_	28.94	($\bar{1}$11)	Monoclinic HfO_2_
	32.18	(111)	Monoclinic HfO_2_
	35.06	(002)	Monoclinic HfO_2_
	50.78	($\bar{2}$20)	Monoclinic HfO_2_
	60.36	(131)	Monoclinic HfO_2_
WO_3_	28.94	(020) *	Tentative/reference-supported WO_3_ assignment
	41.52	(040) *	Tentative/reference-supported WO_3_ assignment
	72.44	(060) *	Tentative/reference-supported WO_3_ assignment
Fe	44.67	(110) *	Tentative bcc α-Fe reference assignment
	65.02	(200) *	Tentative bcc α-Fe reference assignment

* Tentative/reference-supported assignments are shown for WO_3_ and Fe because these features are less clearly resolved than the Bi_2_O_3_, BaSO_4_, and HfO_2_ reflections.

**Table 4:** Photopeak transmission data and linear attenuation coefficients at 661.7 keV (^137^Cs).

Specimen	$I_{0}$	*I*	Thickness (mm)	μ (cm^−1^)	$\sigma_{\mu ,\mathrm{count}}$ (cm^−1^)
Pure cement board	291,694	270,651	6.00	0.1248	0.0044
30% Fe	291,694	268,207	4.85	0.1731	0.0055
30% HfO_2_	291,694	270,800	4.15	0.1791	0.0064
30% WO_3_	291,694	271,165	4.04	0.1806	0.0066
30% Bi_2_O_3_	291,694	271,415	3.95	0.1824	0.0068
30% BaSO_4_	291,694	268,495	4.50	0.1842	0.0059

**Table 5:** Photopeak transmission data and linear attenuation coefficients at 59.5 keV (^241^Am).

Specimen	$I_{0}$	*I*	Thickness (mm)	μ (cm^−1^)	$\sigma_{\mu ,\mathrm{count}}$ (cm^−1^)
Pure cement board	376,739	297,984	6.00	0.3909	0.0041
30% Fe	376,739	275,898	4.85	0.6423	0.0052
30% HfO_2_	376,739	177,558	4.15	1.8127	0.0069
30% WO_3_	390,605	139,626	4.04	2.5464	0.0077
30% BaSO_4_	376,739	115,856	4.50	2.6205	0.0075
30% Bi_2_O_3_	376,739	125,506	3.95	2.7828	0.0083

**Table 6:** Effective mass-normalized attenuation coefficient and derived penetration parameters.

Specimen	Energy (keV)	$\mu_{m}$ (cm^2^ g^−1^)	HVL (cm)	TVL (cm)	MFP (cm)
Pure cement board	661.7	0.0835	5.554	18.450	8.013
Pure cement board	59.5	0.2616	1.773	5.890	2.558
30% Bi_2_O_3_	661.7	0.0884	3.800	12.624	5.482
30% Bi_2_O_3_	59.5	1.3489	0.249	0.827	0.359
30% BaSO_4_	661.7	0.1017	3.763	12.500	5.429
30% BaSO_4_	59.5	1.4470	0.265	0.879	0.382
30% HfO_2_	661.7	0.0912	3.870	12.856	5.583
30% HfO_2_	59.5	0.9230	0.382	1.270	0.552
30% WO_3_	661.7	0.0895	3.838	12.750	5.537
30% WO_3_	59.5	1.2625	0.272	0.904	0.393
30% Fe	661.7	0.1030	4.004	13.302	5.777
30% Fe	59.5	0.3823	1.079	3.585	1.557

**Table 7:** Contextual comparison of linear attenuation coefficients reported near 662 keV for selected cementitious shielding materials and conventional lead as a reference benchmark.

Material/Formulation	Density (g cm^−3^)	μ (cm^−1^)	Condition/Additive	Reference
Pure pressed cement board	1.494	0.1248	Control	Present study
Cement board + 30% Fe	1.680	0.1731	30 wt% Fe	Present study
Cement board + 30% HfO_2_	1.964	0.1791	30 wt% HfO_2_	Present study
Cement board + 30% WO_3_	2.017	0.1806	30 wt% WO_3_	Present study
Cement board + 30% Bi_2_O_3_	2.063	0.1824	30 wt% Bi_2_O_3_	Present study
Cement board + 30% BaSO_4_	1.811	0.1842	30 wt% BaSO_4_	Present study
Ordinary concrete, different grades	2.13–2.39	0.172–0.195	Limestone/sand aggregate	[10]
Ordinary concrete with siltstone replacement	2.05–2.30	0.146–0.160	0–40 wt% siltstone	[17]
Ordinary concrete	–	0.209	No Bi_2_O_3_	[9]
Concrete + 5 wt% bulk Bi_2_O_3_	–	0.213	Bulk Bi_2_O_3_	[9]
Concrete + 5 wt% nano-Bi_2_O_3_	–	0.230	Nano-Bi_2_O_3_	[9]
High-density concrete with ultrafine admixture	>3.70	0.2266	Barite powder + steel slag	[14]
Lead (reference benchmark) *	11.35	≈1.26	Elemental Pb; interpolated NIST data	[16]

* The lead value is an approximate linear attenuation coefficient at 661.7 keV obtained by log–log interpolation of neighboring NIST mass attenuation coefficients and multiplication by the tabulated lead density; it is provided only as a conventional-shield benchmark.

## Data Availability

The original contributions presented in this study are included in the article. Further inquiries can be directed to the corresponding author.
